# Supplementary material for: COmpliance with pandemic COmmands Scale (COCOS): The relationship between compliance with COVID-19 measures and sociodemographic and attitudinal variables
Source: PLoS One. 2022 Jan 19;17(1):e0262698. doi: 10.1371/journal.pone.0262698 (PMC8769316; doi:10.1371/journal.pone.0262698)
Supplement: Attachment — Submitted filename: Response to Reviewers.docx [file pone.0262698.s001.docx]

RESPONSE TO REVIEWERS

Response to the Editor:

1. As the editor suggests, we have reviewed the PLOS ONE's style requirements, and we have carried out several corrections in the manuscript and the title page. Furthermore, we have changed the name of the file.
2. We have reviewed the manuscript and we have made some corrections, to avoid typos and grammatical errors. Furthermore, we have included in the revised manuscript all the information previously removed for blind review.
3. As funding information should not appear in the Acknowledgments section or other areas of the manuscript, we have removed the Acknowledgements section of our manuscript. As suggested by the Editor, we have included our amended statement within our cover letter, in order you change the online submission form on our behalf.
4. We have reviewed our reference list to ensure that is correct and complete. Furthermore, we have introduced the three references suggested by Reviewer 2. On the other hand, we have not cited any paper that have been retracted.

Response to Reviewer 1:

Le agradecemos al revisor/a sus sugerencias en relación al manuscrito, ya que han sido muy útiles para mejorar el artículo. De hecho, a partir de estos comentarios, hemos introducido diversos cambios en el texto. A continuación explicamos estos cambios, y respondemos las diferentes cuestiones que ha planteado.

1. Tal como el revisor ha señalado, la tabla 1 y 2 proporcionaban información redundante, ya que ambas mostraban las medias y desviaciones típicas de los ítems. Por ese motivo, hemos eliminado estos valores de la Tabla 1. Las diferencias en las medias de cuatro de los ítems se debían al redondeo de los decimales. Hemos revisado todos los valores, de tal forma que ahora el redondeo sigue el siguiente patrón: cuando el segundo decimal es igual o inferior a 4, hemos redondeado a la baja, y cuando el segundo decimal es de 5 o más, hemos redondeado al alza. Por otra parte, hemos teniendo en cuenta las sugerencias del revisor y hemos añadido más información en la Tabla 2. Concretamente, esta tabla muestra ahora los valores mínimos y máximos de cada ítem y los coeficientes de asimetría y curtosis.
2. Tal como sugiere el revisor, hemos dejado un apartado exclusivamente para las estadísticas de los ítems y otro apartado para las comparaciones de medias.

Dado que el estudio está centrado en el seguimiento de las medidas preventivas, tan sólo hemos evaluado las diferencias entre hombres y mujeres en el seguimiento de estas medidas y en la opinión sobre la utilidad de las mismas. Estamos de acuerdo en que sería interesante proporcionar también resultados sobre las diferencias de sexo en las otras variables, pero hemos considerado que estos análisis alargarían mucho un artículo que ya es de por sí relativamente largo, cuando estos análisis van más allá de los objetivos planteados inicialmente en el estudio, ya que el resto de variables no se refieren directamente a las medidas preventivas.

3. Le agradecemos al revisor sus comentarios positivos en relación a los procedimientos y resultados de la primera parte del estudio. En relación a la segunda parte, estamos de acuerdo en que la tabla 4 podía resultar confusa, en parte porque algunos de los ítems no estaban incluidos en el apartado Measures, tal como ha señalado el revisor. Por ese motivo, hemos añadido un nuevo párrafo en el apartado Measures, con los ítems que faltaban. También hemos hecho diversos cambios en la Tabla 4 para facilitar su interpretación, y para que resulte más fácil enlazarla con el apartado Measures. Le agradecemos la sugerencia al revisor, dado que consideramos que este apartado resulta ahora más claro que antes.
4. La aplicación que usamos para completar los cuestionarios no permitía dejar ningún ítem en blanco. De hecho, los ítems aparecían de uno en uno, y no se podía pasar al ítem siguiente si el actual estaba en blanco. Por ese motivo, no hay datos perdidos. Por lo tanto, las diferencias en las medias de cuatro ítems en la tabla 1 y 2 no se deben a la existencia de datos perdidos, si no al redondeo de decimales, como hemos explicado anteriormente. Hemos especificado en el artículo que no hay datos perdidos.
5. Tal como el revisor ha planteado, los adolescentes y jóvenes están bien representados en la muestra, probablemente porque son lo que más utilizan las nuevas tecnologías, y por lo tanto son los que más participan en estudios que se realizan de forma online. Pero eso no implica que haya muy pocos adultos con más de 30 años en este estudio, ni que el número de adultos de estas edades sea totalmente insuficiente o inadecuado. Concretamente, en la muestra hay un 28% de adolescentes, un 29% de jóvenes entre los 18 y 29 años, y un 43% de adultos con 30 años o más. Por otra parte, el 31% de la muestra tiene 40 años o más. De hecho, realizamos una ardua tarea de difusión para lograr que la muestra fuera lo más heterogénea posible, y muestra de ello es que en este estudio incluso han participado personas con más de 60 años (70 personas). Sin embargo, a pesar de toda la difusión realizada, el 57% de la muestra tiene menos de 30 años, por lo que hemos reconocido esta limitación en la Discusión.

Response to Reviewer 2:

Le agradecemos al revisor sus comentarios y sugerencias, dado que nos han ayudado a clarificar y mejorar algunos aspectos del artículo. A continuación exponemos los cambios que hemos realizado en el manuscrito y respondemos a las cuestiones planteadas por el revisor.

1. Hemos ordenado mejor los objetivos, de tal forma que ahora parecen en el mismo orden que en los resultados.

2. Tal como dice el revisor, el contenido del ítem de optimismo es general, no se refiere específicamente a la COVID-19. Concretamente, el ítem es el siguiente: “En una escala de 0 a 10, ¿hasta qué punto te consideras una persona optimista?”. Hemos especificado en el texto que el ítem no se refiere a la COVID-19, si no al optimismo en general.

3. Como recomienda el revisor, hemos explicado con más detalle qué medios y entidades se consultaron para obtener información sobre las medidas preventivas recomendadas, incluyendo las citas pertinentes. Concretamente, consultamos las recomendaciones propuestas por la OMS y por el Centers for Disease Control and Prevention, las medidas recomendadas por el Ministerio de Sanidad, Consumo y Bienestar Social y por las comunidades autónomas, y también las medidas incluidas en investigaciones previas. En general, las medidas se engloban en los diferentes ámbitos: uso de la mascarilla, distancia social, higiene de manos, cubrir la nariz y la boca con el codo flexionado o con un pañuelo, cumplimiento de los toques de queda y de las restricciones de movilidad impuestas por las diferentes administraciones, aislamiento en caso de presentar síntomas o de ser diagnosticado de COVID-19, aislamiento en caso de ser contacto de una persona diagnosticada de COVID-19, y otros (evitar fiestas, aglomeraciones, transporte público, dar la mano, viajar sin una razón justificada, compartir bebida y comida con personas con las que no convives, o tocarse los ojos, nariz y boca). Por este motivo, se redactaron ítems de cada una de estas áreas, como puede observarse en la Tabla 1.

Considerando que se partía de diferentes facetas, el revisor pregunta si hubiera tenido sentido plantear algún otro modelo que contemplara estas facetas, como por ejemplo un bifactor. La idea, desde luego, tiene sentido. Sin embargo, los resultados sugieren que estos datos tienen un ajuste excelente al modelo undimensional y, por tanto, que, al extraer el factor común, los residuales virtualmente desaparecen. El modelo bifactor es útil cuando, además de un factor dominante, existen factores de grupo justificables que impiden un buen ajuste del modelo unidimensional. Sin embargo, tratar de extraer factores de grupo a partir de residuales que ya son prácticamente nulos, es un caso de sobre-factorización y no es justificable. Para ver este punto desde una perspectiva más específica, cabe notar que el valor del índice MIREAL es inferior a 0.30. Este índice indica cuál es el promedio de las saturaciones secundarias en sucesivos factores una vez se ha extraído el factor principal. Por tanto, si se intentara extraer adicionalmente un solo factor de grupo (no digamos más de uno) este factor no estaría ni siquiera mínimamente determinado. Por ese motivo, no optamos por la opción de un bifactor. Los resultados sugieren que hay un único factor subyacente a los diferentes ítems y facetas, y por ese motivo no hemos probado otros modelos.

4. Tal como señala el revisor, la tabla 1 y 2 eran redundantes, porque ambas mostraban las medias y desviaciones típicas de los ítems. Por ese motivo, hemos eliminado esta información de la tabla 1. Además, tal como sugiere el revisor, hemos añadido información adicional en la tabla 2: coeficientes de asimetría y curtosis, valores mínimo y máximo en cada ítem, y correlaciones ítem-total corregidas.

5. La fiabilidad se muestra en el texto, concretamente en las líneas 392-394 de la página 15. La frase es la siguiente: Finally, the marginal reliability of the content factor score estimates was *ρ_θθ_* = .92, which can be considered to be quite adequate even for individual assessment purposes”.

6. y 7. Hemos reformulado el párrafo de los objetivos, de tal forma que la parte de la investigación referida a las diferencias de sexo (Tabla 3) está ahora mejor justificada, y más enlazada con el resto del estudio. Por lo tanto, los análisis de la Tabla 3 están ahora bien incluidos en los objetivos, de tal forma que se justifica mejor que se presenten en una tabla propia. Es cierto que esta parte no constituye el objetivo principal ni central del estudio, pero consideramos que era interesante añadirla, dado que estudios previos ya habían mostrado diferencias de sexo en el cumplimiento de las medidas preventivas. Por lo tanto, uno de nuestros objetivos era determinar si estas diferencias también se observaban con el cuestionario COCOS, en población española.

8. Tal como ha solicitado el revisor, hemos aportado la n para cada grupo en la Tabla 4.

9. El revisor pregunta donde se encuentra la variable optimismo en los resultados. Concretamente, la correlación entre optimismo y cumplimiento de las medidas preventivas está en la página 21 del documento, líneas 483-484. La frase es la siguiente: “The correlation between compliance and the item on self-perception as an optimist was not significant (r = .021, p >.05).”

10. Tal como sugiere el revisor, hemos añadido en la discusión que en el futuro se podría desarrollar una versión corta del cuestionario, ya que sería útil en contextos práctico-clínicos. Hemos utilizado las referencias que el revisor había puesto como ejemplo, para justificar la utilidad de una versión más corta del cuestionario.

11. Tal como el revisor ha señalado en relación a la tabla 1, el ítem 8 no se veía bien, ya que la fila no era lo suficientemente ancha como para mostrar todo el texto. Por ese motivo, hemos ampliado esa fila, de tal forma que ahora se puede leer todo el texto.
